# Real-world 12-month outcomes of repeated high-concentration capsaicin patch in chemotherapy-induced peripheral neuropathy: results from the CASPAR study

**DOI:** 10.3389/fonc.2025.1711597

**Published:** 2025-12-04

**Authors:** Michael A. Überall, Rainer Sabatowski, Michael Patrick Lux, Myriam Heine, Lucia Garcia Guerra, Mariëlle Eerdekens, Tamara Quandel

**Affiliations:** 1 Institute of Neurological Sciences, Nuremberg, Germany; 2 Pain Clinic, Department of Anesthesiology and Intensive Care, Medical Faculty “Carl Gustav Carus”, Technical University, Dresden, Germany; 3 Department for Gynecology and Obstetrics, St. Louise Women’s Hospital, Paderborn, St. Josefs Hospital, Salzkotten, St. Vincenz Clinics Salzkotten and Paderborn, Paderborn, Germany; 4 Medical Affairs, Grünenthal GmbH, Aachen, Germany; 5 Medical Affairs, Grünenthal Pharma S.A., Madrid, Spain

**Keywords:** chemotherapy-induced peripheral neuropathy, CIPN, peripheral neuropathic pain, high-concentration capsaicin patch, real-world data, topical treatment, progressive response

## Abstract

**Background:**

Chemotherapy-nduced peripheral neuropathy (CIPN) is a frequent and debilitating complication of cancer therapy, often leading to chronic pain, impaired quality of life (QoL), and reduced daily functioning. This study evaluated the long-term effectiveness and safety of repeated high-concentration capsaicin patch (HCCP) treatment for CIPN using real-world data from the German Pain e-Registry (GPeR).

**Methods:**

In this retrospective, observational, non-randomized cohort study, 169 CIPN patients who received one to four HCCP treatments were followed at 3-monthly intervals (± 2 weeks) over 12 months. Assessed outcomes included average 24-hour pain intensity (API), QoL, sleep, emotional well-being, daily functioning, analgesic use, and safety.

**Results:**

Of 169 patients, 65 received one, 35 two, 25 three, and 44 four treatments. At month 12, patients receiving four treatments showed a marked API reduction on the 0–100 mm VAS scale (from 55.9 mm to 17.3 mm), compared to a minimal change in the one-treatment group (56.9 mm to 53.2 mm). A ≥30% API reduction was achieved in 20.0%, 54.3%, 76.0%, and 97.7% of patients receiving one to four treatments, respectively. Clinically meaningful improvements were also observed in sleep, QoL, emotional distress, and functional capacity, along with a decline in analgesic use. While continued treatment was associated with cumulative benefits, early discontinuation was consistently linked to symptom recurrence. Adverse events were mostly mild, transient, and limited to the application site.

**Conclusion:**

This real-world analysis indicates that repeated HCCP treatment is associated with progressive improvements in pain intensity, QoL, and functional outcomes in patients with CIPN, while maintaining good long-term tolerability.

## 1 Introduction

For many cancer types, chemotherapy is an essential form of treatment. However, given the neurotoxicity of these treatments, there is a risk of developing chemotherapy-induced peripheral neuropathy (CIPN). CIPN often improves after chemotherapy ends. However, depending on the agent and the patient’s history, symptoms can persist, emerge later, or even worsen after treatment (1, 2). Estimates indicate that approximately one third of patients experience chronic pain (≥6 months) following the completion of chemotherapy (3).

CIPN is characterized by pain, tingling, sensory deficits (e.g., numbness), and increased sensitivity to thermal and mechanical stimuli, primarily in the hands and feet, with possible extension to the arms and legs. The underlying pathophysiology, clinical manifestation, severity, and course of CIPN vary considerably. By interfering with numerous aspects of daily life, CIPN can lead to a profound reduction in quality of life (QoL). Sleep and the ability to work are frequently compromised. Furthermore, daily life and activities can be significantly impacted by gait instability and balance disorders, including a tendency to fall and difficulties with fine motor skills. Involvement of autonomic nerve fibers can also affect the function of internal organs, leading to blood pressure instability, gastrointestinal disturbances, or incontinence (4–6). Adding to the impact on the individuals concerned, persistent complaints and periods of inability to work can cause significant socio-economic burden for patients, the cost carriers and society (7).

Treatment options for CIPN primarily aim to alleviate symptoms and improve QoL, typically involving a combination of pharmacologic and non-pharmacologic approaches. While systemic agents such as antidepressants, anticonvulsants, and opioids are frequently used, their efficacy is limited and variable, with systemic adverse effects being a common concern (8–10). Topical treatment with high-concentration (179 mg) capsaicin patch (HCCP) has become a valuable additional option for treating peripheral neuropathic pain of various etiologies, including CIPN (2, 11–17). HCCP is approved in the European Union for all kinds of peripheral neuropathic pain and in the United States for postherpetic neuralgia and diabetic peripheral neuropathic pain of the feet (18, 19).

Meanwhile, there is growing evidence that repeated HCCP treatments can lead to progressive response in patients with a range of peripheral neuropathic pain conditions. Even patients who do not experience adequate relief from the initial HCCP treatment may respond to further treatments, subsequently achieving improvements in pain intensity, sleep quality, and other efficacy measures (20–22). However, data on benefits of repeated HCCP treatment in CIPN patients is still limited (11, 12, 20, 21, 23).

The CASPAR study aims to assess the impact of continued HCCP treatments in patients with various peripheral neuropathic pain indications analyzing real-world data from the German Pain e-Registry (GPeR) (24–26). In this analysis, we focus on the effectiveness and safety of HCCP therapy and the evaluation of progressive benefits in a sub-cohort of patients with CIPN.

## 2 Methods

### 2.1 Study design and patients

This analysis was based on a sub-cohort from the retrospective, observational, non-randomized, multi-cohort study CASPAR (registry number: EUPAS1000000106) and reports unique CIPN sub-cohort data that have not been published elsewhere. Registered patients aged ≥18 years with a clinically confirmed diagnosis of CIPN were eligible for inclusion if they received their first HCCP treatment for CIPN between January 1, 2015, and December 31, 2021. Inclusion further required complete data for the full 12-month period (up to December 31,2022) following the first HCCP treatment, for both patients who continued and those who discontinued HCCP thereafter. Patients with comorbid progressive diseases (e.g., multiple sclerosis, active cancer, end-of-life care) or psychological conditions unrelated to pain (e.g., substance abuse, severe drug dependency) were excluded to reduce confounding effects on pain perception and treatment response. The full set of exclusion criteria has been reported previously (24–26).

**Figure 1:**
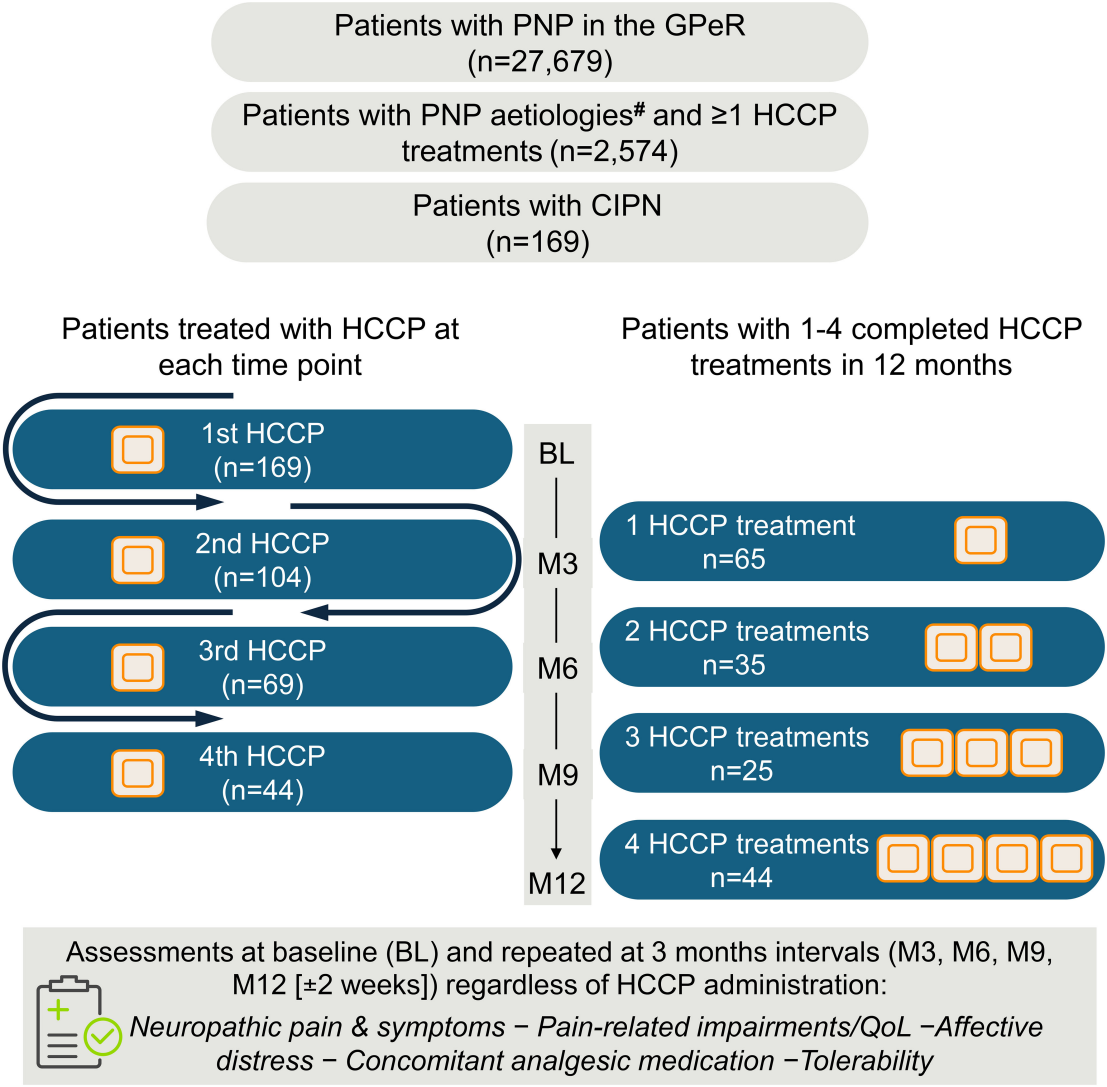
Study design and patient flow. Between January 1, 2015, and December 31, 2021, a total of 27,679 patients with PNP were recorded in the GPeR. Of these, 2,574 were eligible for inclusion in the CASPAR cohort. Among them, 169 patients had CIPN and formed the full analysis set. During the 12-month observation period, patients received between one and four HCCP treatments. The left panel shows the number of patients treated with HCCP at each scheduled 3-month (± 2 weeks) time point (BL, M3, M6, M9). The right panel groups patients according to the total number of HCCP treatments completed during the study period, reflecting early discontinuation: 65 patients received one treatment, 35 received two, 25 received three, and 44 completed four treatments. Clinical assessments were conducted at BL and repeated every three months (± 2 weeks), independent of treatment continuation. ^#^Categorized as postherpetic neuralgia (PHN), peripheral nerve injury (PNI), painful diabetic peripheral neuropathy (PDPN), and others, including chemotherapy-induced peripheral neuropathy (CIPN). BL, baseline; CIPN, chemotherapy-induced peripheral neuropathy; EoS, end of study (Month 12); GPeR, German Pain e-Registry; HCCP, high-concentration capsaicin patch; M, month; PNP, peripheral neuropathic pain; QoL, quality of life.

All patients were evaluated over a period of 12 months. Assessments were made prior to the initial HCCP treatment (baseline) and continued after each additional treatment or immediately preceding the next treatment (three-month ±2 weeks intervals and up to 12 months after the first treatment). In case of HCCP treatment discontinuation, patients were evaluated within ±2 weeks of the initially intended time of retreatment (**Figure 1**).

### 2.2 Data source

The CASPAR study is based on anonymized data collected from January 1, 2015 to December 31, 2021 in the German Pain e-Registry (GPeR), a national web-based pain treatment registry established by the Institute of Neurological Sciences (IFNAP) on behalf of the German Pain Association (27). The registry collects routine data from pain patients through the iDocLive^®^ online documentation platform with the goal of optimizing patient treatment. Patient-entered data comprise pain characteristics, impairments in daily activities and QoL, treatment responses, and other relevant information for personalized patient care. During clinic visits, physicians reviewed and supplemented patient data, such as demographics, diagnosis, history, and medication, to ensure accuracy and guide treatment decisions.

The GPeR employs standardized and validated patient questionnaires recommended by the German Pain Association, the German Pain Society, and the German Pain League (including the German Pain Questionnaire and the German Pain Diary) (27).

CASPAR is registered with the European Network of Centers for Pharmacoepidemiology and Pharmacovigilance in the European Union Electronic Registry of Post-Authorization Studies. In the context of the CASPAR study and in line with the GPeR’s standard operating procedures, anonymized data were temporarily extracted from the registry database for the purposes of healthcare research. This non-interventional study followed the principles of the Declaration of Helsinki and all applicable national regulations. Ethical approval was not required in accordance with national legislation and institutional requirements, as the study analyzed anonymized registry data without direct patient contact or intervention. All patients provided written informed consent for use of their data in the German Pain e-Registry for research purposes. All data analyses in this study were performed independently by the research team without sponsor input or oversight. Data access was restricted to anonymized records, and all interpretations reflect independent academic judgment.

### 2.3 Effectiveness assessments

Effectiveness assessments and definitions of treatment response have been described in detail elsewhere (24–26). Briefly, pain intensity was assessed using the 24-hour Average Pain Intensity (API) on a 0–100 mm Visual Analogue Scale (VAS), with higher scores indicating more severe pain (28). The Pain Intensity Index (PIX) was calculated as the mean of lowest, highest, and average VAS scores within 24 hours. Clinically meaningful treatment response was defined as a ≥30% or ≥50% reduction in API and/or a ≥20 mm decrease on the VAS (10, 29).

Neuropathic symptoms were evaluated using the 7-item painDETECT Questionnaire (PDQ-7), with items rated from 0 (never) to 5 (very strongly), yielding a total score from 0 to 35 (30). Pain-related disability was assessed with the Modified Pain Disability Index (mPDI), where each item is rated from 0 (no impairment) to 100 (complete impairment) (31, 32); the sleep-specific item was analyzed separately (mPDI-6 sleep score).

Emotional distress was measured with the 21-item Depression Anxiety Stress Scale (DASS-21), comprising three subscales with seven items scored from 0 to 3 per item, with domain-specific severity thresholds (normal to extremely severe) (33). Suicidal ideation was assessed via a single item (none/sometimes/frequent).

QoL was evaluated using the Veterans RAND (VR)-12, which provides a norm-based Physical Component Summary (PCS) and a Mental Component Summary (MCS) score (mean=50, SD = 10) (34–36). The Quality-of-Life Impairment by Pain Inventory (QLIP) was used to quantify the pain-related impact on daily activities, emotional well-being, and social participation. The total score ranges from 0 (maximum impairment) to 40 (no impairment); scores ≤20 indicate considerable QoL limitations (37). General well-being was assessed using the Marburg Questionnaire on Habitual Health Findings (MQHHF), total score range 0–35, with lower scores reflecting greater symptom burden (38).

Chronic pain severity was graded using the von Korff Chronic Pain Grading, combining pain intensity, disability, and days of activity limitation into four hierarchical severity levels from low- to high-impact chronic pain (39). Analgesic use (e.g., opioids, antiepileptics, antidepressants) was tracked to assess medication burden over time. Adverse events (AEs) were patient-reported and physician-reviewed; adverse drug reactions (ADRs) were defined as AEs with a possible, probable, or definite relationship to HCCP treatment.

### 2.4 Statistical analysis

Statistical analysis followed previously described methods (24–26). In brief, a stratified analysis by the total number of HCCP treatments (1–4 treatments) over the 12-month follow-up was performed for most analyses. Descriptive analyses for ordinally scaled data (e.g., rankings or numerical scores) used mean, standard deviation (SD), median and 95% confidence intervals (CI). Changes versus baseline were either expressed absolute or relative for scores as well as proportions. Significance was set at p<0.05 (two-sided). Longitudinal changes across all four follow-up visits were assessed using mixed-model repeated-measures analysis of variance (ANOVA). Effect sizes were estimated using Cohen’s d, odds ratios (OR), relative risks, numbers needed to treat or harm (NNT, NNH), and the phi coefficient, where applicable. Cohen’s d was interpreted according to conventional thresholds: small (0.2–0.49), moderate (0.5–0.79), and large (≥0.8). Effect sizes, unlike p-values, quantify the magnitude of an effect independently of sample size and offer a direct indication of clinical relevance (40).

Effectiveness endpoints included changes in API, PIX, PDQ-7, DASS-21, mPDI, VR-12, QLIP, MQHHF and von Korff Chronic Pain Grading with comparisons made to baseline and, where applicable, previous treatment intervals. Safety outcomes included the incidence and nature of ADRs.

## 3 Results

### 3.1 Baseline characteristics

Data analysis comprised 169 CIPN patients. Of these, 65 completed a single HCCP treatment, 35 two, 25 three, and 44 patients had four HCCP treatments during the 12-month study period (**Figure 1**). Baseline characteristics are summarized in **Table 1**. In brief, mean (SD) age was 61.9 (13.8) years and 55.6% were female. Furthermore, mean (SD) pain duration was 3.5 (3.5) years. At baseline, average API and PIX scores were comparable at 57.2 (19.4) and 57.3 (16.5), respectively, on a 0–100 VAS scale. Most patients (71.6%) were classified as von Korff grade 4. In total, 92.3% were grade 3 or 4, indicating high disability with moderate to severe limitations. On average, patients were unable to engage in their usual activities for 58.2 days in the preceding 3 months, corresponding to more than half of all days in this period. The majority of patients exhibited symptoms of CIPN in the feet (40.8%) and hands (28.4%). Based on the PDQ-7, the most frequently reported moderate to very strong neuropathic symptoms were thermal hyperalgesia (71.6%), burning sensations (63.9%), allodynia (61.6%), and numbness (61.6%). The VR-12 revealed greater impairment in physical than in mental health-related QoL with mean (SD) scores of 27.5 (7.1) and 38.6 (21.1), respectively. A substantial impact on overall impairment in QoL was reported by 84.0% of patients according to the QLIP. Severe or extreme sleep disturbances were documented in 33.7% of patients, corresponding to a mean (SD) VAS score of 58.0 (22.5) for the mPDI item 6. Overall well-being, as measured by the MQHHF, was markedly reduced in 62.7% of patients. The psychological burden was considerable, with the DASS-21 indicating moderate to severe or extremely severe depression, anxiety, and stress in 58.5%, 47.9%, and 54.4% of patients, respectively. Suicidal ideation was present in 30.2% of patients at least occasionally. Prior to baseline, patients had consulted a mean of seven different physicians/specialties and had received eight neuropathic pain or adjuvant medications. At baseline, they were on average taking four such medications, most commonly high-potency opioids (73.4%), antiepileptics (67.5%), and antidepressants (81.7%) (**Table 1**).

**Table 1:** Demographics and baseline characteristics of trial participants.

Patient characteristics	CIPN population (n=169)
Age, years, mean ± SD (min; max)	61.9 ± 13.8 (21, 91) 59.2% ≤65 years
Gender, female, n (%)	94 (55.6)
Pain duration, years, mean ± SD (min; max)	3.5 ± 3.5 (0; 12)
Baseline pain intensity, 0−100 mm VAS	
Average 24-hour Pain Intensity (API), mean ± SD (min; max)	57.2 ± 19.4 (19; 100)
Pain Intensity Index (PIX), mean ± SD (min; max)	57.3 ± 16.5 (30.7; 100)
Von Korff pain severity grading, n (%)	
Grade 1 (low disability − low pain intensity)	0 (0)
Grade 2 (low disability − high pain intensity)	13 (7.7)
Grade 3 (high disability − moderately limiting)	35 (20.7)
Grade 4 (high disability − severely limiting)	121 (71.6)
Number of days usual activities could not be performed due to neuropathic pain in the past 3 months, mean ± SD (min; max)	58.2 ± 32.5 (0; 92)
Modified Pain Disability Index (mPDI), 0−100 mm VAS	
mPDI total score, mean ± SD (min; max)	64.3 ± 18.3 (29.3; 97.7)
mPDI-6 sleep score, mean ± SD (min; max)	58.0 ± 22.5 (21; 100)
mPDI-6 sleep disability category, n (%)	
None or mild (0−30)	30 (17.8)
Moderate (31−50)	40 (23.7)
Strong (51−70)	42 (24.9)
Severe/extreme (>70)	57 (33.7)
Veterans RAND 12-item Health Survey (VR-12), based on population average score of 50	
VR-12 Physical Component Score (VR-12 PCS), mean ± SD (min; max)	27.5 ± 7.1 (14.1; 50.4)
VR-12 Mental Component Score (VR-12 MCS), mean ± SD (min; max)	38.6 ± 12.1 (19.1; 68.5)
Depression Anxiety and Stress Scale (DASS-21) (%)	
Depression, moderate/strong/severe or extremely severe (score ≥7)	99 (58.5)
Anxiety, moderate/strong/severe or extremely severe (score ≥6)	81 (47.9)
Stress, moderate/strong/severe or extremely severe (score ≥10)	92 (54.4)
Quality of Life Impairment by Pain (QLIP), patients with clinically relevant impairment (sum score <20), n (%)	142 (84.0)
Overall well-being (MQHHF), strong/severe (sum score ≤10), n (%)	106 (62.7)
Suicidal ideation (sometimes or frequent), n (%)	51 (30.2)
Neuropathic pain phenotype (7-item painDETECT^®^ Questionnaire, PDQ-7), each item scored 0–5	
Burning, moderate/strong/very strong (score ≥3), n (%)	108 (63.9)
Prickling, moderate/strong/very strong (score ≥3), n (%)	103 (61.0)
Allodynia, moderate/strong/very strong (score ≥3), n (%)	104 (61.6)
Pain attacks, moderate/strong/very strong (score ≥3), n (%)	89 (52.6)
Thermal hyperalgesia, moderate/strong/very strong (score ≥3), n (%)	121 (71.6)
Numbness, moderate/strong/very strong (score ≥3), n (%)	104 (61.6)
Pressure-evoked pain, moderate/strong/very strong (score ≥3), n (%)	104 (61.5)
Pain localization (treatment area), n (%)	
Arms	16 (9.5)
Hands	48 (28.4)
Pelvic area	5 (3.0)
Legs	31 (18.3)
Feet	69 (40.8)
Number of specialties/physicians involved per patient, mean ± SD (min; max)	6.7 ± 1.3 (3; 9)
Previous neuropathic pain or adjuvant medications^1^, mean ± SD (min; max)	8.0 ± 2.4 (3; 14)
Current neuropathic pain or adjuvant medications, mean ± SD (min; max)	3.9 ± 1.5 (1; 8)
Patients receiving neuropathic pain or adjuvant medications, n (%)	
High-potency opioid analgesic^2^	124 (73.4)
Antiepileptic drugs	114 (67.5)
Antidepressant drugs	138 (81.7)

^1^Prior to first visit. ^2^High-potency opioid analgesics, i.e. buprenorphine, fentanyl, hydromorphone, morphine, oxycodone, pethidine, and piritramide. CIPN, chemotherapy-induced peripheral neuropathy; MQHHF, Marburg Questionnaire on Habitual Health Findings; SD, standard deviation; VAS, Visual Analogue Scale.

### 3.2 Neuropathic pain and symptoms

**Figure 2:**
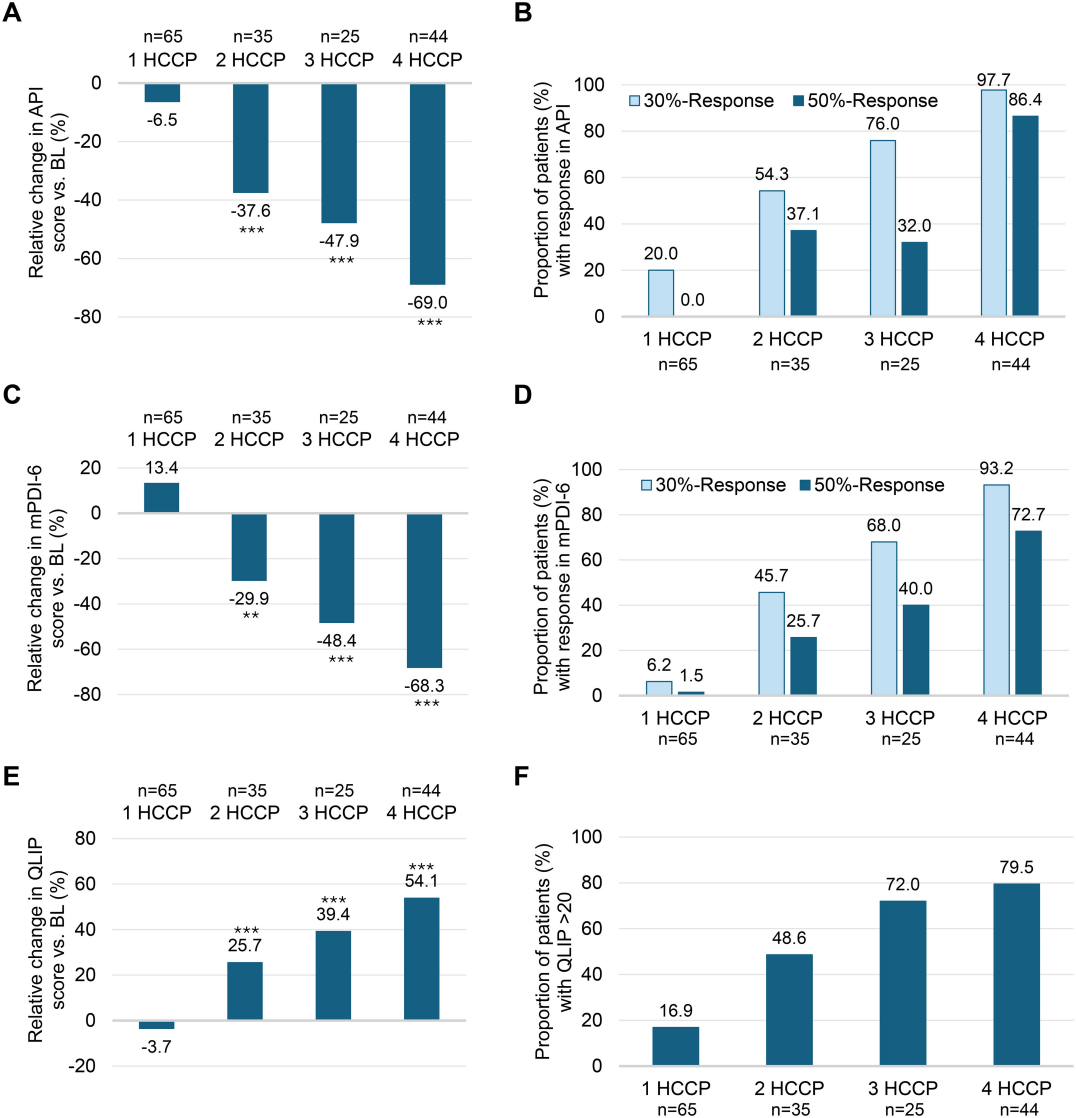
Twelve-month changes in pain intensity, sleep disturbance, and QoL impairment related to pain, stratified by the number of completed HCCP treatments (one to four). Relative changes in **(A)** API (↓ improvement), **(C)** mPDI-6 (↓ improvement), and **(E)** QLIP scores (↑ improvement) from baseline to month 12 in patient subgroups receiving one, two, three, or four HCCP treatments. Response rates (≥30% and ≥50%) based on **(B)** API and **(D)** mPDI-6, as well as **(F)** the proportion of patients without clinically relevant QoL impairment (QLIP >20) after 12 months depending on the number of HCCP treatments received. **p<0.01 *vs* BL; ***p<0.001 *vs*. BL. API, Average Pain Intensity in the preceding 24 hours; BL, baseline; HCCP, high-concentration capsaicin patch; mPDI-6, Modified Pain Disability Index, subscale #6 (mPDI-sleep score); QLIP, Quality of Life Impairment by Pain; QoL, quality of life.

Based on the total number of HCCP treatments during the 12-month study period, patients were stratified into four subgroups (one to four HCCP; **Figure 1**). After the initial treatment, all subgroups exhibited comparable reductions in pain intensity, with API scores decreasing by 18.8% to 24.4% from baseline (p<0.05 to p<0.001). With continued treatment, subgroup differences became apparent, with greater improvements observed in patients receiving a higher number of HCCP applications (**Supplementary figure 1A**). At month 12, patients who received a single treatment showed a mean (SD) reduction in API from 56.9 mm (19.6) to 53.2 mm (21.7) (−6.5%; p=0.316), whereas in patients receiving four treatments, the API decreased from 55.9 mm (19.1) to 17.3 mm (16.3) (−69.0%; p<0.001) (**Figure 2A**). At the same timepoint, 20.0% of patients who received only one HCCP treatment achieved a ≥30% reduction in API compared to baseline, whereas this applied to 97.7% of those who received four treatments. A ≥50% reduction in API was not achieved by any patient in the one-treatment group, while 86.4% of those receiving four treatments reached this threshold (**Figure 2B**).

In addition to analgesic effects, repeated HCCP treatment was associated with sustained improvements in neuropathic symptom severity, as assessed by the PDQ-7 questionnaire. At month 3, patients who ultimately received either one or four treatments exhibited comparable symptom reductions across PDQ-7 domains with total scores decreasing by 16.9% and 16.5% from baseline, respectively (p<0.001). However, by month 12, divergent patterns emerged: patients who received only a single HCCP application showed a deterioration in symptoms, with PDQ-7 total scores increasing from 20.0 (SD 3.1) at baseline to 25.0 (SD 6.9), whereas those who completed four treatments maintained their improvement, with scores decreasing from 20.1 (SD 3.3) to 16.4 (SD 2.7) (**Table 2**). The greatest symptom reductions were observed for allodynia (−40.2%, p<0.001), burning sensation (−22.0%, p=0.017), and prickling (−20.2%, p=0.086). More modest changes were noted for pressure-evoked pain (−14.3%, p=0.133), numbness (−12.5%, p=0.225), and pain attacks (−11.7%, p=0.316), with the smallest reduction seen in thermal hyperalgesia (−9.9%, p=0.276). Between-group comparisons at month 12 yielded effect sizes (Cohen’s d) consistent with clinically meaningful benefits of repeated treatment, independent of statistical significance: large effects were observed for prickling (d=1.245) and numbness (d=0.925); moderate-to-large effects for pressure-evoked pain (d=0.766) and allodynia (d=0.733); and moderate effects for burning sensation (d=0.650) and pain attacks (d=0.507) (**Table 2**).

**Table 2:** PDQ-7 sum score and individual symptom item scores at baseline, and after three and twelve months, respectively, in patients who completed either one or four HCCP treatments.

	1 HCCP treatment (n=65)					4 HCCP treatments (n=44)					
	BL	Month 3	% Change* (BL *vs*. month 3)	Month 12	% Change* (BL *vs*. month 12)	BL	Month 3	% change* (BL *vs*. month 3)	Month 12	% Change* (BL *vs*. month 12)	Effect size (Cohen’s d) 1 *vs*. 4 HCCP (month 12)
PDQ-7 sum score, mean (SD); range 0-35	20.0 (3.1)	16.6 (2.6)	-16.9 *(p<0.001)*	25.0 (6.9)	25.0 *(p<0.001)*	20.1 (3.3)	16.8 (2.7)	-16.5 *(p<0.001*)	16.4 (2.7)	-18.5 *(p<0.001)*	1.644 (large)
PDQ-7 item scores (range: 0-5)											
Burning	2.7	2.2	-16.2 *(p=0.029)*	3.2	20.2 *(p=0.062)*	2.8	2.2	-22.0 *(p=0.017)*	2.2	-22.0 *(p=0.017)*	0.650 (moderate)
Prickling	2.9	2.4	-18.8 *(p=0.028)*	4.0	35.4 *(p<0.001)*	2.6	2.1	-20.2 *(p=0.086)*	2.1	−20.2 *(p=0.086)*	1.245 (large)
Allodynia	2.7	2.0	-27.5 *(p=0.001)*	3.0	9.8 *(p=0.412)*	2.9	2.1	-26.8 *(p=0.002)*	1.7	-40.2 *(p<0.001)*	0.733 (moderate-to-large)
Pain attacks	2.4	1.3	-12.0 *(p=0.241)*	3.4	39.6 *(p=0.001)*	2.9	2.6	-11.7 *(p=0.316)*	2.6	-11.7 *(p=0.316)*	0.507 (moderate)
Thermal hyperalgesia	3.3	2.7	-18.7 *(p=0.014)*	3.8	15.7 *(p=0.079)*	3.2	2.9	-9.2 *(p=0.314)*	2.9	-9.9 *(p=0.276)*	0.590 (moderate)
Numbness	2.8	2.5	-9.9 *(p=0.246)*	3.8	37.9 *(p<0.001*	2.7	2.4	-12.5 *(p=0.225)*	2.4	-12.5 *(p=0.225)*	0.925 (large)
Pressure-evoked pain	3.2	2.7	-14.6 *(p=0.056)*	3.8	18.9 *(p=0.036)*	3.0	2.6	-14.3 *(p=0.133)*	2.6	-14.3 *(p=0.133)*	0.766 (moderate-to-large)

*Positive/negative values in the “% change” columns indicate worsening/improvement of symptoms relative to baseline. Cohen’s d values (1 *vs*. 4 HCCP) represent effect sizes based on between-group differences in 12-month scores. Values of 0.2–0.49 indicate small, 0.5–0.79 moderate, and ≥ 0.8 large effects. HCCP, high-concentration capsaicin patch; PDQ-7, 7-item painDETECT^®^ Questionnaire; SD, standard deviation; BL, Baseline.

### 3.3 Psychological and functional Quality-of-Life outcomes

Improvements in sleep-related impairment (mPDI-6) followed a similar progressive response pattern according to the number of HCCP treatments received, as observed for pain intensity (**Supplementary Figure 1B**). At month 12, sleep disturbance scores decreased by 68.3% from baseline in patients who received four treatments (p<0.001), whereas a 13.4% increase was observed in those who discontinued treatment after the initial treatment (p= 0.072; **Figure 2C**). The proportion of patients achieving ≥30% and ≥50% improvement in sleep scores reached 93.2% and 72.7%, respectively, in the four-treatment group, compared to 6.2% and 1.5% among those treated once (**Figure 2D**). Pain-related QoL, assessed by the QLIP score, also differed according to the number of HCCP treatments received during the study period. Patients who completed four HCCP treatments showed a mean relative score improvement of 54.1% (p<0.001), compared to a decline of 3.7% (p=0.397) in those treated only once; accordingly, 79.5% of patients in the four-treatment group reported no clinically relevant impairment (QLIP >20), whereas this applied to only 16.9% of patients who had received a single treatment (**Figures 2E, F**).

At month 12, patients who received four HCCP treatments showed the greatest improvements in emotional well-being, as assessed by the DASS-21. Statistically significant reductions in mean scores from baseline were observed for depression (8.5 to 4.4; p<0.001), anxiety (5.7 to 3.0; p=0.002), and stress (10.2 to 5.3; p<0.001). In contrast, patients who received only one treatment exhibited increases in mean scores for depression (10.0 to 11.9), anxiety (6.6 to 8.1), and stress (11.1 to 12.9) (data not shown). This pattern was further reflected in categorical severity analyses: the proportion of patients classified as moderately to extremely affected was substantially lower in the four-treatment group, with 27.2% for depression (cut-off ≥7), 13.6% for anxiety (≥6), and 15.9% for stress (≥10), compared to 76.9%, 55.3%, and 63.1%, respectively, among patients who had received only a single treatment (**Supplementary Table 1**). Notably, severe or extreme symptoms markedly increased across all domains in the one-treatment group, while virtually disappearing in the four-treatment group (0.0% for depression and stress; 4.5% for anxiety; **Supplementary Figure 2**). In parallel, the proportion of patients reporting suicidal ideation also decreased over the 12-month period. At baseline, 30.2% of all patients reported experiencing suicidal thoughts at least occasionally (**Table 1**). At month 12, a reduction was first noted in the three-treatment group (−8.0%), and suicidal ideation was no longer reported in the group that received four consecutive HCCP treatments (corresponding to a −27.2% change from baseline). In addition, general well-being improved most substantially in patients receiving four HCCP treatments, with the mean MQHHF global score reflecting general well-being increasing by 80.4% from baseline (**Supplementary Table 1**).

**Figure 3:**
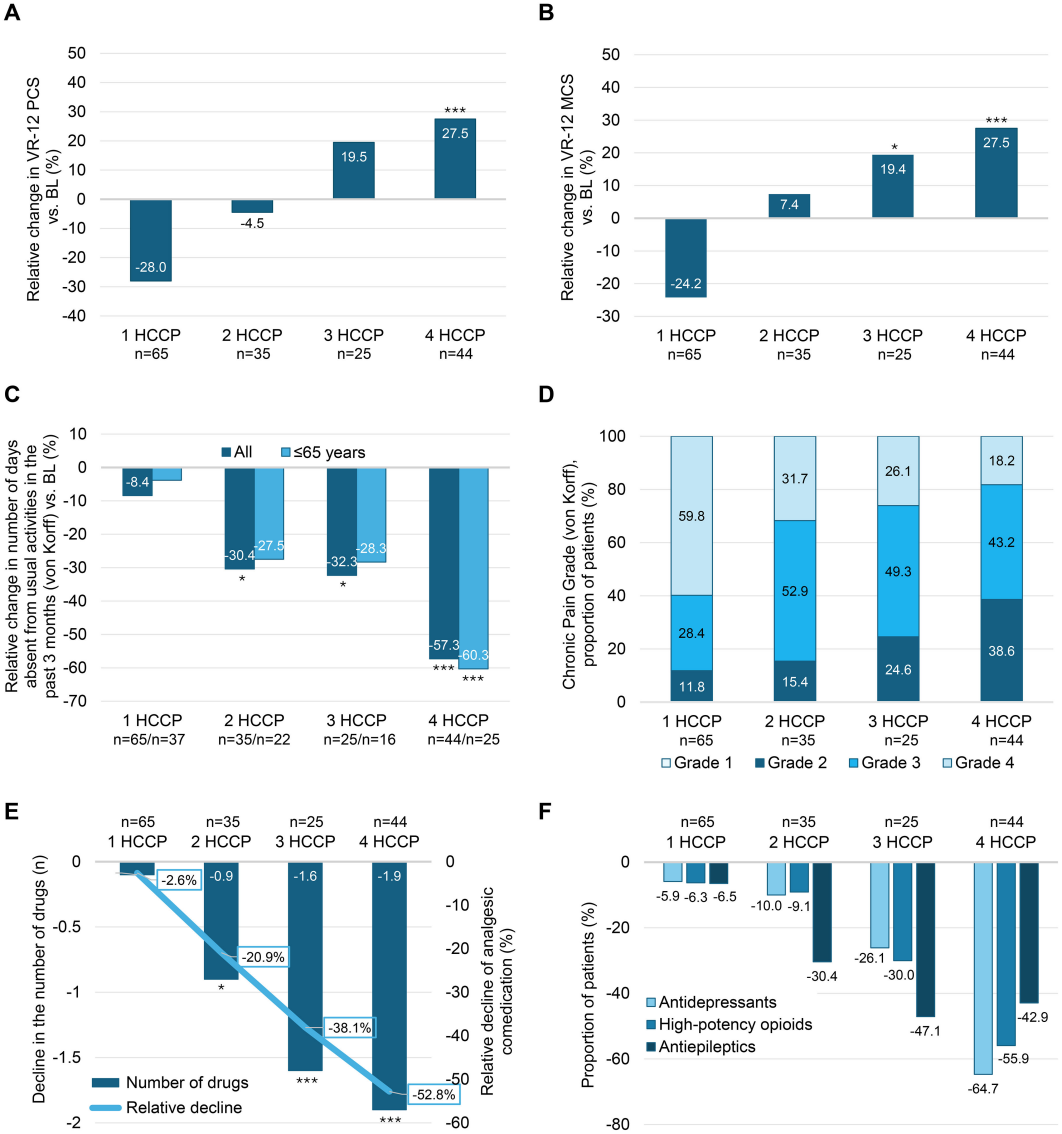
Twelve-month changes in health outcomes (physical and mental health, activity impairment, severity of chronic pain, and analgesic comedication use), stratified by the number of HCCP treatment (one to four). Relative change in physical **(A)** and mental **(B)** health according to the VR-12 PCS and MCS scores (↑ improvement), respectively. **(C)** Relative changes in the number of days absent from usual activities, based on von Korff assessment. **(D)** Changes in pain severity grading (von Korff). **(E)** Change in the number or proportion (%) of systemic analgesics/co-analgesics used. **(F)** Proportion of patients with antidepressant, high-potency opioid or antiepileptic comedication. All data refer to the 12-month follow-up and are stratified by the number of consecutive HCCP treatments completed. *p<0.05 *vs* BL; ***p<0.001 *vs*. BL. HCCP, high-concentration capsaicin patch; MCS, Mental Component Score; PCS, Physical Component Summary; VR-12, Veterans RAND 12-item Health Survey.

Physical health status, measured by the VR-12 PCS score, showed a similar treatment-dependent pattern: relative changes ranged from –28.0% after one treatment (reflecting deterioration) to +27.5% after four (**Figure 3A**). A comparable trend was observed for the VR-12 MCS score with relative changes at study end ranging from −24.2% to +27.5% (**Figure 3B**). After three and four HCCP treatments, the mean VR-12 MCS reached/exceeded the normative value of 50, which represents the average mental health status in the general population with mean (SD) scores of 50.1 (14.5) and 51.8 (16.6), respectively (data not shown). Furthermore, the number of days that could not be spent on usual activities due to neuropathic pain in the past 3 months declined by more than half compared to baseline in patients who received four HCCP treatments. This effect was observed in the overall population and in the subgroup aged ≤65 years, which comprised 59.2% of the study cohort (**Figure 3C**). In addition, the proportion of patients with lower pain severity grades (von Korff grade <4) increased with the number of HCCP treatments. At baseline, 28.4% of patients were classified below grade 4 (**Table 1**); this proportion rose markedly at month 12 to 81.8% among those who ultimately received four treatments (**Figure 3D**).

### 3.4 Comedication

By month 12, the average number of concomitant analgesic agents used per patient had progressively declined, with relative reductions ranging from −2.6% after one HCCP treatment to −52.8% after four treatments (**Figure 3E**). A similar trend was observed across individual systemic drug classes. Compared to baseline, the proportion of patients receiving concomitant antidepressants, high-potency opioids, and antiepileptic drugs decreased by 26.1%, 30.0%, and 47.1%, respectively, after three treatments, and by 64.7%, 55.9%, and 42.9%, respectively, after four treatments (**Figure 3F**).

### 3.5 Responder conversion with repeated treatment

**Figure 4:**
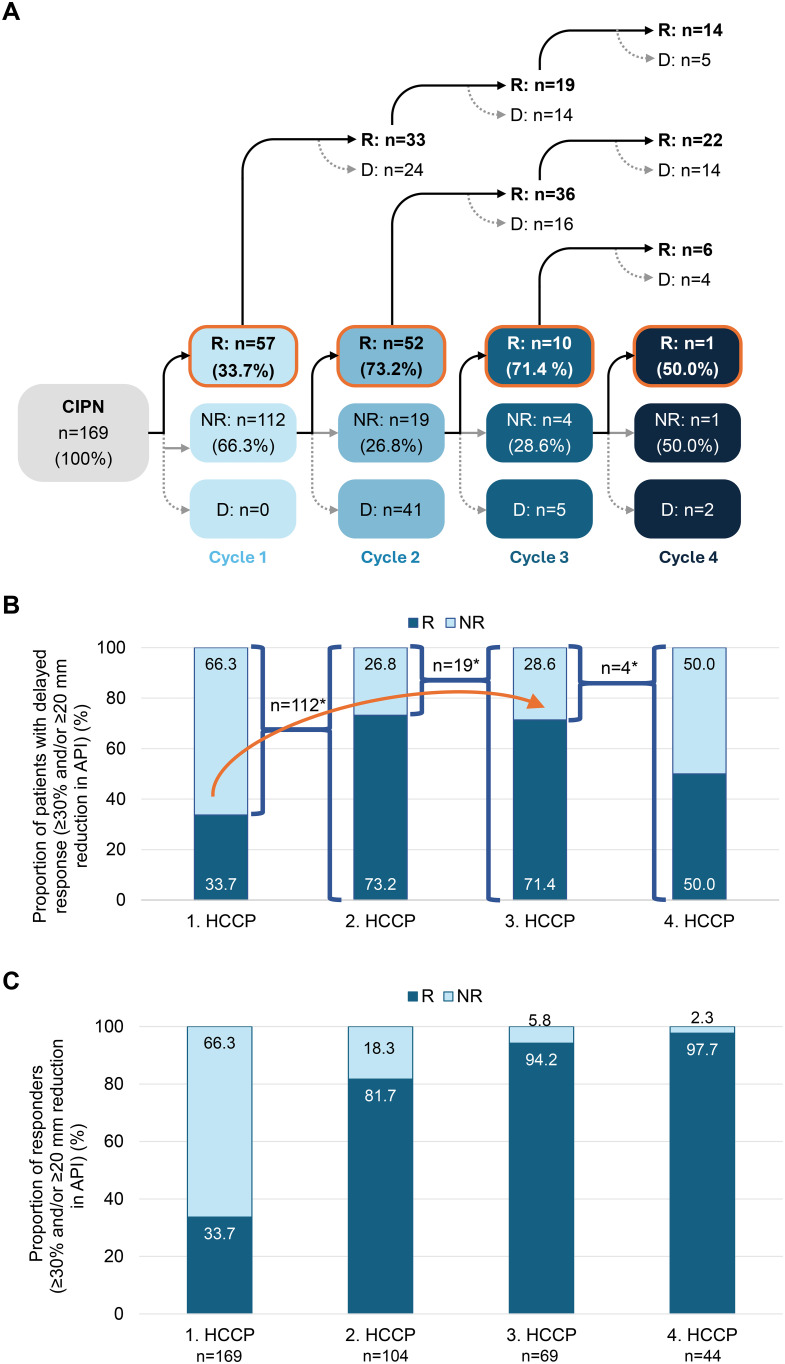
Conversion of initial non-responders to responders with successive HCCP treatments. **(A)** Patient flow following the first, second, third, and fourth HCCP treatments, with classification as responder (R) or non-responder (NR) based on a clinically meaningful reduction in average API, defined as a ≥20 mm and/or ≥30% improvement on the VAS. **(B)** Proportion of responders after the first HCCP treatment and among initial non-responders who received subsequent treatments. The arrow indicates the progressive conversion of initial non-responders to responders with successive HCCP treatments. In A and B, percentages refer to the number of non-responders treated further at each respective time point. **(C)** Percentage of responders 3 months after the first to fourth HCCP treatment (includes all patients who received treatment at each time point). *: n without D. API, Average Pain Intensity in the preceding 24 hours; CIPN, chemotherapy-induced peripheral neuropathy; D, treatment discontinuation; HCCP, high-concentration capsaicin patch; NR, non-responders; R, responders; VAS, Visual Analogue Scale.

Among the 169 patients initially treated, 66.3% (n=112) did not achieve a clinically meaningful response defined as a reduction in pain intensity of ≥20 mm and/or a ≥30% improvement in API (VAS) and were classified as non-responders. With repeated HCCP treatment, a substantial proportion of these patients could be converted into responders. After the second treatment, 73.2% (n=52/71) of retreated non-responders achieved response, followed by 71.4% (n=10/14) after the third and 50.0% (n=1/2) after the fourth treatment (**Figures 4A, B**). None of the patients who achieved a clinically meaningful response and continued HCCP treatment lost their responder status during the study (**Figure 4A**, upper part).

The progressive conversion contributed to a steady increase in the overall cumulative response rate across treatment cycles. After the second treatment, 81.7% (n=85/104) of patients had achieved a clinically meaningful response. This proportion further increased to 94.2% (n=65/69) following the third treatment and reached 97.7% (n=43/44) after the fourth treatment (**Figure 4C**).

### 3.6 Adverse drug reactions and reasons for treatment discontinuation

ADRs were observed in 55.6% of patients after the first treatment and in 57.7% after the second. The frequency of ADRs decreased with subsequent HCCP treatments, affecting 46.4% of patients at the third and 29.5% at the fourth patch application. The most frequently reported application-site reactions were pain, erythema, and burning sensation (**Supplementary Table 2**). After one HCCP treatment, these events were of mild or moderate intensity in 85.2%, 85.5%, and 85.0% of patients, respectively; following four treatments, all occurrences were classified as mild or moderate. ADRs occurred on average within 1.2 to 1.6 days after application and led to discontinuation of therapy in 5.9% of the exposed patients after one treatment, in 7.7% after two, in 1.4% after three, and in 4.5% after the fourth treatment. Other documented reasons for discontinuation included lack of effectiveness (14.8% to 15.9%), a combination of ADRs and lack of effectiveness (4.7% to 9.1%), and causes not further specified (13.0% to 31.8%) (data not shown).

## 4 Discussion

CIPN remains a significant burden in oncology, with limited effective and well-tolerated treatment options (41). This real-world analysis of GPeR data in a well-characterized cohort of patients with CIPN provides valuable insights into baseline characteristics and longitudinal outcomes under routine clinical conditions. During the 12-month follow-up, patients received between one and four HCCP treatments. Repeated HCCP treatment was associated with progressive reductions in pain intensity, accompanied by improvements in sleep quality, emotional well-being, and a reduced use of concomitant analgesics. In addition, pain-related QoL and daily functioning improved during treatment. These benefits were greatest for patients who received all four HCCP treatments but were lost if treatment was stopped early.

A clinically meaningful response, defined as a ≥30% reduction in 24-hour API and/or a ≥20 mm reduction in VAS score, was observed in 71.4% of patients who had not responded to the first or second HCCP treatments but subsequently received a third application. This finding suggests a delayed onset of therapeutic effect in certain patients. Although the small sample size warrants caution, similar patterns were observed in the overall CASPAR population (n=2,574) across different neuropathic pain conditions: Among the 1,582 initial non-responders, 919 (74.1%) achieved a clinically meaningful pain response after the second HCCP treatment and 171 (77.0%) after the third (manuscript in preparation). In addition, the progressive increase in response rates across treatment cycles, together with the absence of response loss with continued HCCP treatment, supports the hypothesis of a cumulative therapeutic effect and is in line with previous observations across multiple neuropathic pain etiologies, including CIPN, painful diabetic peripheral neuropathy, postherpetic neuralgia, and post-traumatic nerve injury (12, 13, 20–22, 42). Moreover, symptom recurrence upon treatment discontinuation, as observed in our cohort, aligns with findings from the same CASPAR study including patients with painful diabetic peripheral neuropathy and neuropathic pain after trauma or surgery (24–26). From a clinical perspective, these data support the consideration to evaluate treatment benefit after at least three treatment cycles, particularly in patients with an initially insufficient response as recommended in the summary of product characteristics (SmPC) for HCCP since 2023 (18).

The mechanism underlying the progressive response observed with repeated HCCP treatment remains speculative. Recent studies suggest that capsaicin-induced defunctionalization of TRPV1-expressing nociceptive fibers may be followed by structural and functional regeneration, which has been shown to correlate with pain relief (11, 43–45). Repeated HCCP treatments may enhance capsaicin-induced nerve regeneration by triggering successive cycles of defunctionalization and reinnervation, gradually restoring more physiological nociceptive signaling and thereby contributing to the cumulative clinical benefit. The extent and rate of nerve repair, however, may vary among patients depending on factors such as the severity of nerve injury, duration of neuropathy, metabolic status, and individual neuroplastic capacity, potentially explaining why a meaningful clinical response becomes evident only after the second or third application in some patients.

Repeated HCCP treatment was also associated with improvements in specific neuropathic symptoms commonly associated with CIPN. According to PDQ-7 assessments, the greatest relative reductions were observed for allodynia, burning sensations, and prickling, while thermal hyperalgesia and numbness showed more modest changes over time. Notably, patients who discontinued HCCP treatment after a single treatment exhibited deterioration across all assessed neuropathic symptom domains. At month 12, between-group comparisons (1 HCCP *vs*. 4 HCCP) revealed moderate to large effect sizes in all seven PDQ-7 domains, with the strongest effects observed for prickling (d=1.245) and numbness (d=0.925). These findings are consistent with results from the QUCIP study in breast cancer patients with CIPN (12) and support the potential of repeated HCCP treatment to provide sustained and clinically meaningful relief across a broad spectrum of neuropathic symptoms, including sensory deficits.

CIPN has a well-documented impact on emotional well-being, often mediated by impairments in daily functioning, sleep quality, and work capacity (4–6). In line with this, a substantial proportion of patients in our study exhibited clinically relevant symptoms of depression, anxiety, and stress on the DASS-21 scale at baseline. Similarly, low baseline VR-12 scores for physical and mental health reflected a considerable burden of CIPN. With repeated HCCP treatment, these psychological and functional impairments improved substantially, particularly among patients who completed all four treatment cycles, whereas worsening of symptoms was observed in those who discontinued early. Furthermore, assessment of impairment in everyday activities using the von Korff scale showed a marked decrease in days absent from usual activities. Again, the most pronounced improvements were observed after four HCCP treatments, with reductions of 57.3% in the overall cohort and 60.3% in the working-age subgroup compared to baseline. Taken together, these findings point to potential benefits of the treatment beyond analgesic effects, including improvements in pain-related QoL and functional limitations, although a causal relationship cannot be established.

At baseline, most patients were prescribed multiple systemic pain medications, including high-potency opioids, antiepileptic drugs and antidepressants. During the course of HCCP treatment, the use of these medications declined substantially. After four treatment cycles, the overall analgesic burden was reduced by 52.8%, with marked decreases in the proportion of patients receiving antidepressants (−64.7%), high-potency opioids (−55.9%), and antiepileptics (−42.9%). This is a clinically relevant outcome, given the well-documented risks of adverse effects and dependency associated with these drug classes (10), which may in turn also contribute to impaired QoL. The findings are also consistent with previous studies in neuropathic pain populations showing reduced systemic analgesic use following HCCP therapy (22, 46, 47) and are further supported by similar outcomes in other subgroups of the present CASPAR study (24–26). Notably, patients tend to prefer topical therapies such as HCCP due to their favorable safety profile and lower treatment burden than with alternative oral therapies (48, 49).

The safety profile of HCCP in this cohort was consistent with prior clinical trials and real-world evidence, and no new safety signals emerged during the study period (50). While the observed decline in ADRs from 55.6% after the first treatment to 29.5% after the fourth may indicate improved tolerability, it should be interpreted with caution due to the decreasing number of patients over time. Importantly, all ADRs were localized to the application site, with pain, erythema, and burning sensations being the most frequently reported. These reactions were generally mild and transient, regardless of the number of treatments. This tolerability profile of HCCP is consistent with its pharmacological properties. Systemic absorption after topical administration is minimal, with plasma concentrations generally below 10 ng/mL and declining rapidly after patch removal. When used according to the approved instructions, treatment-related reactions are generally mild, transient, and confined to the application site (18).

The interpretation of our study data is subject to certain limitations. As with any observational, non-prospective, and non-controlled study, causal relationships cannot be established, and reverse causality cannot be ruled out, i.e. patients who experienced benefit from initial treatment may have been more likely to receive subsequent treatments. Furthermore, selection bias related to physicians’ treatment decisions and patient characteristics cannot be excluded. However, patients were enrolled only after the independent clinical decision to initiate HCCP treatment had been made.

Due to the structure of the GPeR registry, information on the specific chemotherapeutic agents responsible for CIPN was not available. This limits the interpretation of the data, as potential heterogeneity of treatment response related to different chemotherapy agents could not be analyzed. Notably, previous findings suggest that platinum salt-induced CIPN may be associated with lower response rates following HCCP treatment (20). Despite these limitations, a key strength of the study is the standardized assessment of all outcome parameters at regular intervals, regardless of the number of HCCP treatments received. This enabled consistent analysis across all patients, including those who discontinued treatment early, yielding valuable insights into outcomes after early discontinuation and following repeated treatment.

## 5 Conclusion

This real-world study shows that repeated HCCP treatment is associated with progressive improvements in neuropathic pain and related symptoms, with positive effects on sleep, emotional well-being, functional capacity, and QoL in patients with CIPN. These benefits were sustained only with continued treatment, whereas effects of a single application diminished over time. Consistent with existing literature (12, 22), the data support the need for at least three treatment cycles to achieve optimal therapeutic response. HCCP appears to be a well-tolerated, effective, and health-economically viable treatment option for patients with CIPN who require sustained symptom relief with minimal systemic burden.

## Data Availability

The raw data supporting the conclusions of this article will be made available by the authors, without undue reservation.
