# Supplementary material for: Real-world 12-month outcomes of repeated high-concentration capsaicin patch in chemotherapy-induced peripheral neuropathy: results from the CASPAR study
Source: Front Oncol. 2025 Dec 4;15:1711597. doi: 10.3389/fonc.2025.1711597 (PMC12711474; doi:10.3389/fonc.2025.1711597)
Supplement: Supplementary file 1 [file DataSheet1.pdf]

SUPPLEMENTAL MATERIAL

Real-world 12-month outcomes of repeated high-concentration capsaicin patch in chemotherapy-induced peripheral neuropathy: results from the CASPAR study

Michael A. Überall[1], Rainer Sabatowski[2], Michael Patrick Lux[3], Myriam Heine[4], Lucia Garcia Guerra[5], Mariëlle Eerdekens[4], Tamara Quandel[4]

[1] Institute of Neurological Sciences, Nuremberg, Germany

[2] Pain Clinic, Department of Anaesthesiology and Intensive Care, Medical Faculty "Carl Gustav Carus", Technical University, Dresden, Germany

[3] Department for Gynecology and Obstetrics, St. Louise Women's Hospital, Paderborn, St. Josefs Hospital, Salzkotten, St. Vincenz Clinics Salzkotten & Paderborn, Paderborn, Germany

[4] Medical Affairs, Grünenthal GmbH, Aachen, Germany

[5] Medical Affairs, Grünenthal Pharma S.A., Madrid, Spain

*** Correspondence:**
Dr. Tamara Quandel
Tamara.quandel@grunenthal.com

Supplemental Figure 1

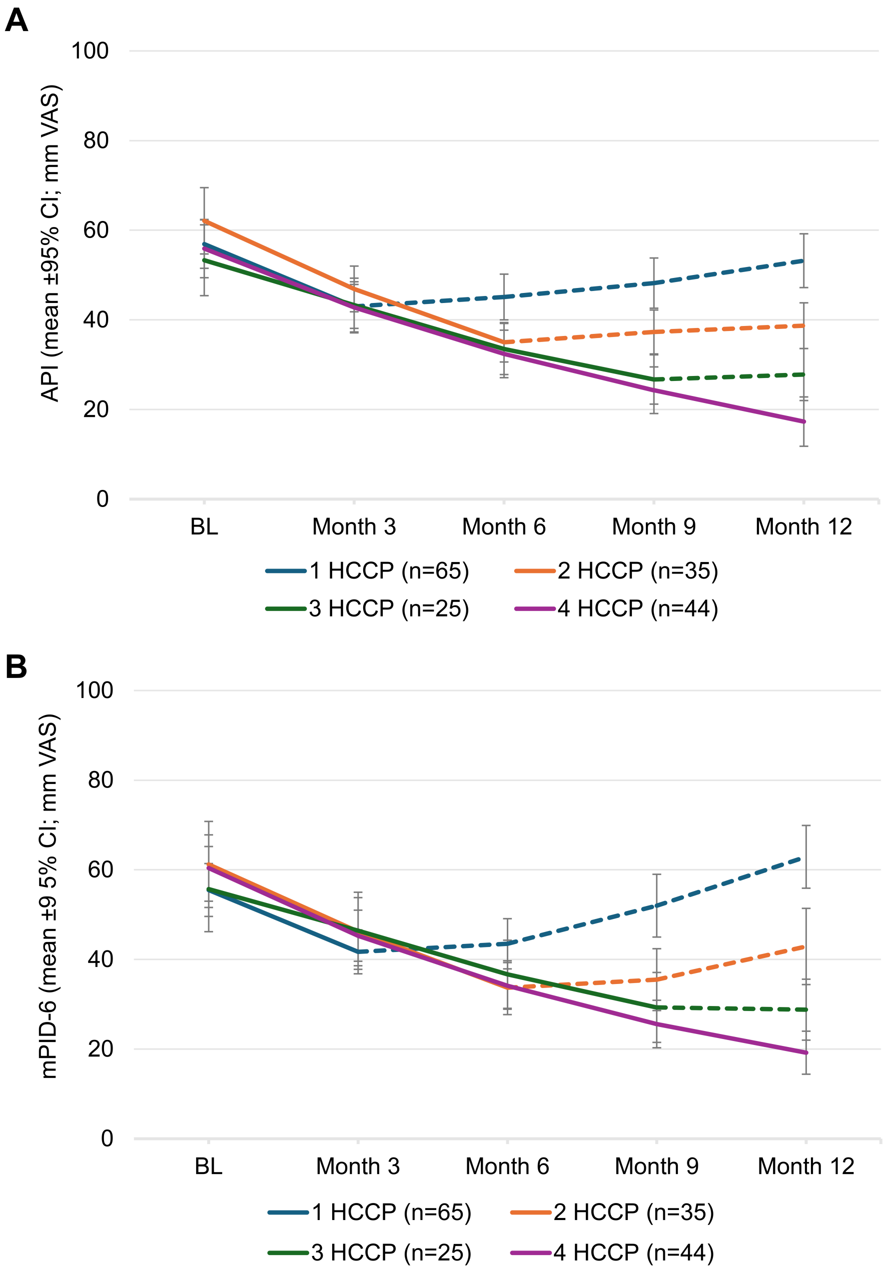

Supplemental Figure 1: Changes in pain intensity (API) and sleep disturbance (mPDI-6) over 12 months by number of HCCP treatments across all treatment locations. (**A**) Average 24-hour pain intensity (API), (**B**) sleep disturbance (mPDI-6). *API: average 24-hour pain intensity; CI: confidence interval; HCCP: high-concentration capsaicin patch; mPDI: Modified Pain Disability Index, subscale #6 (mPDI-sleep score); VAS: visual analogue scale.*

This is a provisional file, not the final typeset article

Supplemental Table 1: Twelve-month changes in affective distress and emotional health, stratified by number of HCCP treatments (one vs. four).

Assessment	Consecutive HCCP treatments	Baseline	Month 12	Absolute (relative) difference	p value
Depression (DASS-21-D; patients with moderate/strong/severe or extreme symptoms), n (%)#	1 (n=65)	45 (69.3)	50 (76.9)	+5 (+7.6)	0.323
	2 (n=35)	22 (62.9)	24 (68.5)	+2 (+5.6)	0.615
	3 (n=25)	9 (36.0)	7 (28.0)	-2 (-8.0)	0.544
	4 (n=44)	23 (52.3)	12 (27.2)	-11 (-25.1)	0.017*
Anxiety (DASS-21-A; moderate/strong/severe or extreme), n (%)#	1 (n=65)	32 (49.3)	36 (55.3)	+4 (+6.0)	0.482
	2 (n=35)	19 (54.2)	17 (48.6)	-2 (-5.6)	0.632
	3 (n=25)	13 (52.0)	7 (28.0)	-6 (-24.0)	0.083
	4 (n=44)	17 (38.6)	6 (13.6)	-11 (-25.0)	0.008**
Stress (DASS-21-S; moderate/strong/severe or extreme), n (%)#	1 (n=65)	38 (58.4)	41 (63.1)	+3 (+4.7)	0.590
	2 (n=35)	22 (62.8)	18 (51.5)	-4 (-11.3)	0.334
	3 (n=25)	10 (40.0)	4 (16.0)	-6 (-24.0)	0.059
	4 (n=44)	22 (50.0)	7 (15.9)	-15 (-34.1)	<0.001***
Suicidal ideation (sometimes/frequent), n (%)†	1 (n=65)	22 (33.8)	22 (33.8)	0 (no change)	1.000
	2 (n=35)	12 (34.3)	12 (34.3)	0 (no change)	1.000
	3 (n=25)	5 (20.0)	3 (12.0)	-2 (-8.0)	0.440
	4 (n=44)	12 (27.2)	0 (0)	-12 (-27.2)	<0.001***
MQHHF (global score), mean (SD)‡ ↑ improvement	1 (n=65)	7.3 (5.9)	5.6 (5.5)	-1.6 (-22.5)	0.109
	2 (n=35)	10.9 (8.4)	11.7 (8.1)	+0.7 (+6.8)	0.712
	3 (n=25)	9.4 (7.0)	13.9 (10.9)	+4.4 (+47.0)	0.100
	4 (n=44)	8.2 (5.8)	14.8 (11.1)	+6.6 (+80.4)	<0.001***

#p value: none/mild vs. moderate/strong/severe or extreme. †p value: none vs. sometimes/frequent. ‡p value: comparison with baseline. *p<0.05, **p<0.01, ***p<0.001.

DASS-21-A/D/S: Depression Anxiety and Stress Scale-21-Anxiety/Depression/Stress; HCCP: high-concentration capsaicin patch; MQHHF: Marburg Questionnaire on Habitual Health Findings; SD: standard deviation.

Supplemental Figure 2

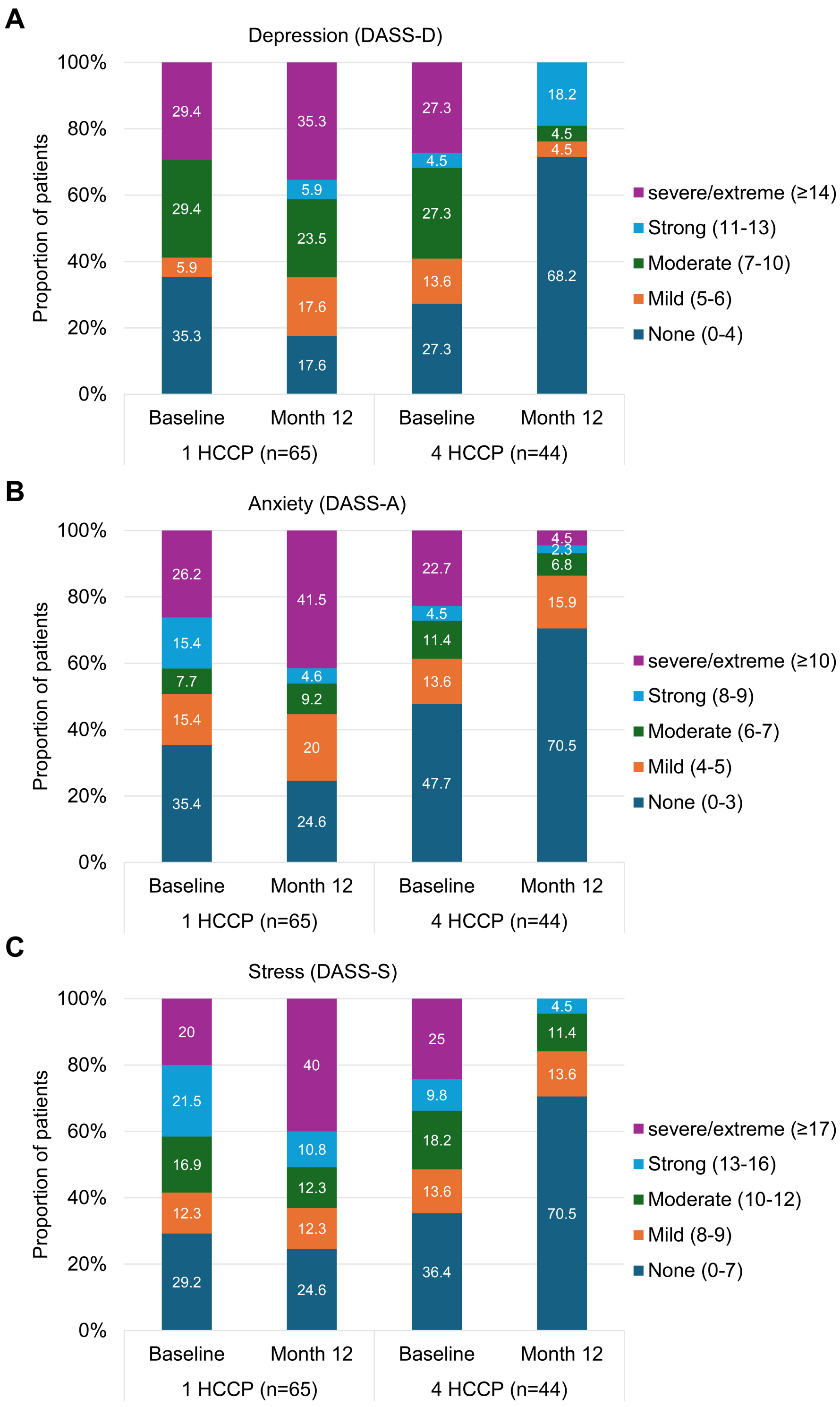

Supplemental Figure 2: Changes in the severity of depression, anxiety, and stress symptoms from baseline to month 12 in patients receiving one versus four HCCP treatments. Severity of depression (**A**), anxiety (**B**), and stress (**C**), as assessed using the DASS-21 self-report scale. Shown are the proportions of patients reporting no, mild, moderate, strong, or severe/extreme symptoms at month 12 following one or four HCCP treatments. *DASS-21: 21-item depression, anxiety and stress scale, HCCP: high-concentration capsaicin patch.*

This is a provisional file, not the final typeset article

Supplemental Table 2: Adverse drug reactions (ADRs)

	1. HCCP (n=169)	2. HCCP (n=104)	3. HCCP (n=69)	4. HCCP (n=44)
**Patients with ADR (%)**	**55.6**	**57.7**	**46.4**	**29.5**
**Discontinuation of treatment due to ADR, patients (%)**	**5.9**	**7.7**	**1.4**	**4.5**
**Spectrum of ADRs, n (%) ***				
Pain	47.9	49.0	37.7	18.2
Erythema	40.8	47.1	30.4	22.7
Burning sensation	11.8	6.7	8.7	2.3
Pruritus	6.5	9.6	11.6	2.3
Hyperaesthesia	5.9	11.5	5.8	9.1
Swelling/edema	8.9	8.7	5.8	2.3
Warmth	7.1	7.7	7.2	0
Paraesthesia	4.1	7.7	10.1	0
Dryness	3.6	2.9	1.4	2.3
Vesicles	6.5	8.7	1.4	2.3

*adjusted percentages for patients exposed to treatment.

ADR: adverse drug reaction; HCCP: high-concentration capsaicin patch.
